# HINT, a code for understanding the interaction between biomolecules: a tribute to Donald J. Abraham

**DOI:** 10.3389/fmolb.2023.1194962

**Published:** 2023-06-07

**Authors:** Glen E. Kellogg, Anna Marabotti, Francesca Spyrakis, Andrea Mozzarelli

**Affiliations:** ^1^ Department of Medicinal Chemistry and Institute for Structural Biology, Drug Discovery and Development, Virginia Commonwealth University, Richmond, VA, United States; ^2^ Department of Chemistry and Biology “A Zambelli”, University of Salerno, Fisciano (SA), Italy; ^3^ Department of Drug Science and Technology, University of Turin, Turin, Italy; ^4^ Department of Food and Drug, University of Parma and Institute of Biophysics, Parma, Italy

**Keywords:** hydrophatic interactions, LogP, HINT, protein–ligand, protein–protein, protein–DNA complexes, water thermodynamics

## Abstract

A long-lasting goal of computational biochemists, medicinal chemists, and structural biologists has been the development of tools capable of deciphering the molecule–molecule interaction code that produces a rich variety of complex biomolecular assemblies comprised of the many different simple and biological molecules of life: water, small metabolites, cofactors, substrates, proteins, DNAs, and RNAs. Software applications that can mimic the interactions amongst all of these species, taking account of the laws of thermodynamics, would help gain information for understanding qualitatively and quantitatively key determinants contributing to the energetics of the bimolecular recognition process. This, in turn, would allow the design of novel compounds that might bind at the intermolecular interface by either preventing or reinforcing the recognition. HINT, hydropathic interaction, was a model and software code developed from a deceptively simple idea of Donald Abraham with the close collaboration with Glen Kellogg at Virginia Commonwealth University. HINT is based on a function that scores atom–atom interaction using LogP, the partition coefficient of any molecule between two phases; here, the solvents are water that mimics the cytoplasm milieu and octanol that mimics the protein internal hydropathic environment. This review summarizes the results of the extensive and successful collaboration between Abraham and Kellogg at VCU and the group at the University of Parma for testing HINT in a variety of different biomolecular interactions, from proteins with ligands to proteins with DNA.

## 1 Introduction

Proteins play many functions in living systems as carriers, enzymes, antibodies, receptors, hormones, mechanical support, and storage. In essentially all these functions, proteins interact with small molecules, metals, and other proteins or peptides. Whenever an interaction occurs between two or more molecules, the recognition is dictated by three key elements: i) complementarity in shape, ii) complementarity in interacting moieties, and iii) free energy of binding. There is also a fourth element that deals with time, i.e., how rapidly molecules see each other and how long they stay together. In some cases, this is a few milliseconds, and in other cases, they remain associated for their entire life before degradation.

**FIGURE 1:**
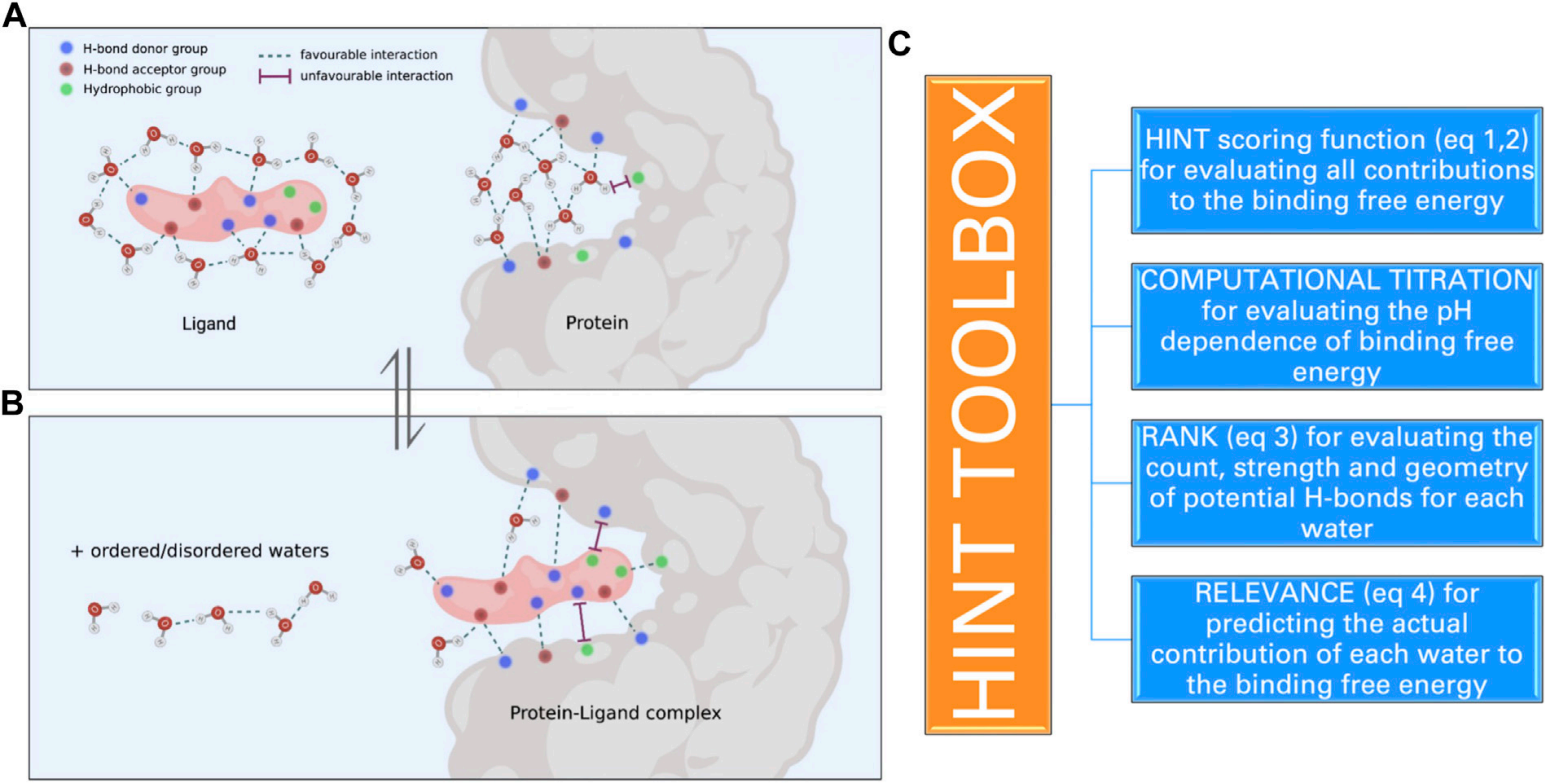
Schematic representation of the molecular events that take place when a ligand binds to a protein site: **(a)** several interactions between ligand polar and apolar groups and protein sidechain residues are formed, and **(b)** water molecules within the active site and bound to the ligand are released into the solvent. These interactions determine the strength of the protein–ligand complex and are computationally evaluated by HINT. **(C)** The toolboxes of HINT for the evaluation of ligand–protein interactions, including water molecules.

Recognition is based on structural and chemical complementarity (Figures 1A,B). Structural complementarity might be obtained upon induced fit (Koshland, 1958; Cozzini et al., 2008) or via a selection among an ensemble of conformations characterized by small energetic differences (Ma et al., 1999). Before and after an interaction, the energy landscape is modified, favoring the conformation that better matches the interacting pair of molecules. This process is driven by energy contributions arising from the determinants at the interface of the partner molecules. Ionic–ionic, polar–ionic, polar–polar, and apolar–apolar hydrogen bonds and hydrophobic interactions contribute to the overall energetics of the recognition. The degree of energetic contributions depends on geometric parameters, such as distance, and the dielectric constant of the medium where interacting groups are localized. Thus, a detailed and precise prediction of the affinity between two molecules can most confidently be obtained when the three-dimensional structure of the complex is known at high resolution and when a quantum mechanical analysis has generated a complete electronic description of the environment. This is a quite challenging goal that has yet to be met, except in toy systems (Yilmazer and Korth, 2016; Cavasotto and Aucar, 2020; Kirsopp et al., 2021). While robust and accurate prediction of the strength of the interaction is complex, the experimental determination of the affinity is often easier but can lack the context of “visualizing” the specific interactions involved. However, for many purposes, the capability of predicting affinities of a complex might actually direct experimental work, such as in the development of potential drugs via structure-based or ligand-based methods. Many efforts have been devoted to the prediction of affinities between proteins and small ligands, proteins, or nucleic acids, and many thoughtful reviews have been published (Ajay and Murcko, 1995; Cozzini et al., 2004; Foloppe and Hubbard, 2006).

Here, we will focus on a very simple approach that exploits both experimental and computational information to generate a score of the interaction that is directly related to the free energy of binding (Figure 1C). This approach was called HINT, which stands for hydropathic interactions (Kellogg and Abraham, 2000). It was developed by a collaboration between the inspired medicinal chemist Donald Abraham and his talented colleague Glen Kellogg at Virginia Commonwealth University (VCU). Abraham had a long-standing interest in the “hydrophobic effect” and its importance in quantitative structure–activity relationship (QSAR) and protein structure. He had, in fact, collaborated with Al Leo of Pomona College in an early article attempting to unify the understanding of LogP (and hydrophobicity) between medicinal chemists and those structural biologists predicting protein secondary structure based on sidechain polarity (Abraham and Leo, 1987). The core concept of HINT, as envisioned by Abraham, was that there was rich thermodynamic information encoded in LogP, and unlocking it would provide insight into more interaction phenomena than simple Newtonian physics-based molecular mechanics force fields. Kellogg joined Abraham at VCU in 1989 and fleshed out this concept by building a new, essentially *de novo*, modeling system that extended the connection matrix-based CLOG-P system of Hansch and Leo (1979) (and the Abraham and Leo enhancements) to the 3D world of structural biology. Thus, formulas for calculating atomistic LogPs were derived that retained the uniquely chemistry-aware features of the CLOG-P system, which itself relied on many thousands of careful measurements of organic and drug-like compounds. Additionally, methods for very quickly estimating or looking up solvent-accessible surface areas (SASAs) for atoms in small molecules, protein residues, and nucleotide bases were programmed (Kellogg and Abraham, 2000). Last, a “distance function” for representing the range effect of hydropathy was determined from some experimental observations indicating an exponential decay (Israelachvili and Pashley, 1982). The first and most obvious application of HINT was in calculating interaction scores between biomolecular species using the atomistic LogPs, atomistic SASAs, and this exponential decay distance function.

Inspired by the pioneering work of David Weininger at Daylight CIS and other researchers, the HINT program was rewritten in the late 1990s as a collection of object-oriented toolkit functions to enable the more facile creation of new application programs exploiting the HINT interaction model (Kellogg et al., 2005). The program is available as a set of toolkit functions by request from Glen Kellogg. The core functions were fully integrated into versions of the Sybyl program but do not, otherwise, have a turnkey application.

A major event in the history of HINT was the development of a very long-term collaboration between VCU’s Abraham and Kellogg and Andrea Mozzarelli, Pietro Cozzini, and several exceptional students at the University of Parma. This collaboration and the numerous exciting results we found are reported in the following paragraphs. It should be noted that many of the innovations of the HINT model were inspired by the various interesting projects that the VCU and University of Parma teams carried out together.

## 2 HINT: definition and applications

### 2.1 The HINT model

#### 2.1.1 Algorithms and code

The core of the HINT code is the assignment to each atom of a factor *a* that is derived from a LogP library coupled with algorithms to appropriately parse this information, where LogP is the partition function of an atom between water and 1-octanol. These two media were selected as representative of the polar and apolar environments within a protein. The atom-type LogP library was adapted from the CLOG-P approach (Hansch and Leo, 1979) with extensions (Abraham and Leo, 1987). HINT counts either positive or negative contributions for each individual atom–atom interaction based on their hydropathic properties. By summing up these atomic contributions, an overall score is obtained. As LogP is a thermodynamic parameter, the HINT score is directly related to the free energy of complex formation. More explicitly, the interaction between two atoms, namely, *i* and *j*, is the product of their atom factors, called partial log P_o/w_ (*a*_*i*_) and solvent-accessible surface area (*S*_*i*_):

$$b_{ij}=a_{i}\;S_{i}\;a_{j}\;S_{j}\;f\left(r_{ij}\right),$$ (1)

where *f(r*_*ij*_ *)* represents a function of the distance between the two atoms, *i* and *j*. The atomistic *S*_*i*_ parameters are applied to represent “exposure” of that atom for interaction with atoms in other molecules. Atom–atom distances are obtained either from the three-dimensional structure of the complex or from a model generated by docking procedures or homology modeling. The higher the resolution (or reliability) of the structures, the more precise the prediction (see Section 3.3). Consequently, the total interaction *B* between two molecules is calculated by the (double) sum over all atom–atom interactions:

$$B=\sum \sum b_{ij}.$$ (2)

A key feature of HINT is the exploitation of the experimentally determined LogP values, thus avoiding complex equations and approximations present in most of the other methods developed for predicting protein–ligand interactions. The other conceptual advantage of using LogP is that this parameter provides an overall representation of the energetics of the encounter process between two molecules, without dividing the energetics into specific enthalpic contributions, such as electrostatic bonds, hydrogen bonds, and van der Waals bonds, a procedure that is not thermodynamically legitimate (Dill, 1997). In addition, and quite relevant, LogP implicitly includes the hydrophobic contribution generated by the change in th3e number of water molecules surrounding the interacting molecules before and upon the complex formation, i.e., the entropic contribution to the free energy (Figures 1A, B). This contribution, which might be significant when apolar molecules interact, is usually not counted by most other programs or roughly approximated by calculating the accessible surface areas in contact between interacting molecules.

#### 2.1.2 Why is HINT different?

The motivation behind HINT was to minimize the “modeling” and extract interaction information and guidance as much as possible from the experiment—in this case, LogP. The measurement of LogP for a small molecule takes place in an environment that has gross similarities to that in which biology takes place, with one solvent (water) commingling with another (1-octanol) that is a stand-in for lipids and membranes. Nevertheless, a few tricks had to be applied, e.g., to make the Hansch and Leo *fragment* constants *atomistic* and to properly sign polar interactions (both Brønsted–Lowry acids and bases are polar) (Kellogg et al., 1992; Kellogg and Abraham, 2000).

While quantitative aspects of programs like HINT are often used for comparative purposes, e.g., in dock scoring, virtual screening, and even LogP prediction, we have always tried to emphasize the qualitative value of HINT. Most importantly, HINT “thinks” like a medicinal chemist—perhaps Donald Abraham in particular—with respect to its language: *hydrogen bonds*, *Lewis acids and bases*, *hydrophobic interactions*, *favorable*, *unfavorable*, *solvation*, and *desolvation.* Thus, HINT’s numerical or graphical output is always very interpretable, and it includes all biomolecular interactions to some degree, as they *are* all holistically encoded in LogP. HINT is, however, not necessarily as accurate as specialized software that focuses on specific phenomena, such as electrostatics, with finely tuned algorithms and parameters. It is, thus, difficult, if not impossible, to directly quantitate HINT’s relative performance compared to other tools in a meaningful way: first, because there are no other tools in that space and, second, because “improved understanding” is not a definable metric.

However, to illustrate the quality of HINT code prediction for the strength of protein and nucleotide complexes, it was tested in many different types of interacting molecules and compared with experimentally determined dissociation constants. The overall results are remarkable, given the simplicity of the code and the speed of calculations. More importantly, virtually all of our studies produced results that suggested or proved new principles of biomolecular interaction. In the next sections, we summarize several cases where HINT has been applied.

## 3 HINT applied to the evaluation of protein–ligand interactions

Many proteins function via interactions with small ligands that exhibit a wide range of features covering most of the chemical space. For example, hemoglobin binds oxygen, which is a neutral molecule, intracellular hormone/vitamin receptors bind mostly apolar ligands, such as estrogens and retinoids, and proteases bind polar–apolar polypeptide chains. This is made possible by sites where ligands bind that possess architectures dictated by the amino acids composing the pocket or site. Since members of the amino acid set are endowed with large differences in the polarity/apolarity of their sidechains, they can accommodate ionic and hydrophobic ligand interactions. In addition, the complementarity between the host (protein) and guest (ligand) may be obtained by optimized conformational changes of either one or the other of the interacting partners to obtain a “perfect” fit or, in a different view, by the selection of the protein and ligand conformations that perfectly match by steric and chemical complementarity. The net result is the formation of a binary complex characterized by an equilibrium between free and ligand-bound molecules regulated by a dissociation constant. The lower the value of the dissociation constant, the higher the match, i.e., the higher the number and overall strength of the interactions.

In order to evaluate how effectively HINT mimics the energetics of biological processes that involve the encounter of proteins and small ligands, we carried out a series of investigations exploring many of the variables that control the strength of protein–ligand interaction. These variables are as follows: i) polarity/apolarity of ligands and/or protein active sites; ii) roles of ligand- and protein-bound water before and upon complex formation; iii) ionization state of ligand moieties and amino acid lateral chains contributing to shape the active sites, i.e., computational titration; iv) orientation of hydrogen atoms bound to polar residues and involved in H-bonds, i.e., the rank toolbox, and v) their actual contribution to the binding free energy, i.e., the relevance toolbox (*vide infra*) (Figure 1C).

As structural information derived by X-ray crystallography is of paramount relevance to HINT analysis, as well as most of the codes aimed at the prediction of protein–ligand energetics, the quality of the three-dimensional structures significantly impacts the confidence of the predicted scores.

### 3.1 Free energy of interactions

We first evaluated by HINT the free energy of binding for 53 protein–ligand complexes formed by 17 proteins of known three-dimensional structure and characterized by different active site polarities (Marabotti et al., 2000; Cozzini et al., 2002). This analysis was carried out without the contribution of bound water molecules to the binding energy. A successive analysis considered such contributions (see Section 4.1) (Cozzini et al., 2004; Fornabaio et al., 2004; Kellogg et al., 2004). To demonstrate that HINT was able to correctly predict the interaction energy between protein and ligands, independently of ligand and protein active site polarity, the selected protein’s active sites vary from very apolar, such as retinol-binding proteins, where the hydrophobic contribution and, consequently, the entropic contribution to the binding energy is predominant, to polar, such as penicillopepsin, where the free energy of interaction is mainly associated with contributions from Coulombic interaction between polar or ionizable residues. Another key feature of the analyzed set of protein–ligand complexes is represented by the large range of binding strength that varies over nine orders of magnitude, with ΔG ranging from −2 to −15 kcal/mol, as calculated by either inhibition constants or dissociation constants (Cozzini et al., 2002).

**FIGURE 2:**
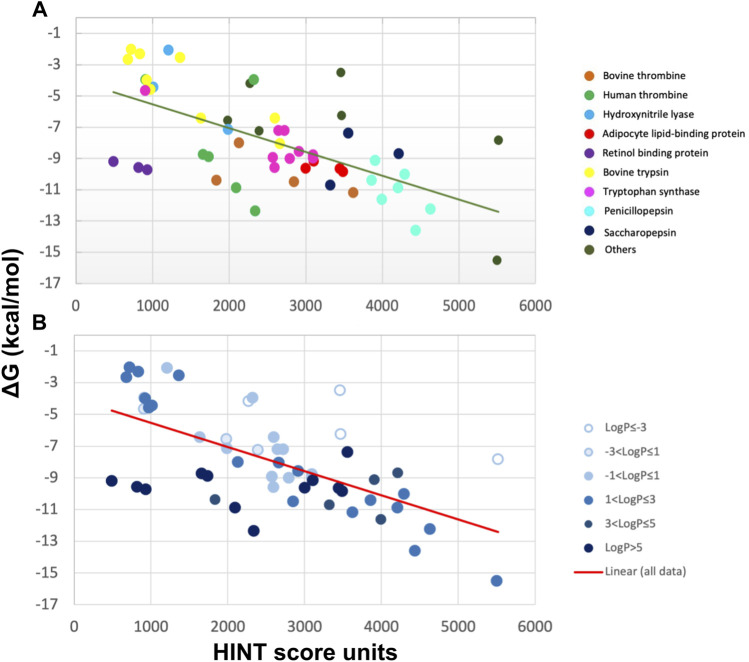
Correlation between experimental ∆G and HINT score units for 53 protein–ligand complexes from more than 10 distinct proteins **(A)** characterized by a wide range of ligand polarity as indicated by their LogP values **(B)** (Cozzini et al., 2002). The line is the best least-squares fit.

HINT scores for the protein–ligand complexes were plotted against the experimental binding affinity, obtaining a remarkably good correlation with a standard error of 2.6 kcal/mol, which translates into a prediction of affinity within about two orders of magnitude (Figure 2). Even better standard errors (1 kcal/mol) were obtained within single sets of specific ligand–protein complexes, such as trypsin, thrombin, and tryptophan synthase. Given the polarity heterogeneity of the 53 protein–ligand complexes, the prediction was very good.

### 3.2 Effects of pH on interactions

A key requirement for obtaining good correlation between computational and experimental data, thus predicting binding affinity for ligands with known three-dimensional complex structures, is the homogeneity between the pH at which binding affinities are measured in solution and the pH at which structures have been determined. In fact, frequently, solution data are determined at pH values quite different from crystal structure medium. As a result, the protonation state of groups might be incorrectly attributed (or modeled), thus affecting the scores. A telling story is represented by an analysis of the binding affinity of penicillopepsin–ligand complexes measured at pHs 3.5, 4.5, and 5.5 and by three-dimensional structures determined at the same pH values. HINT score prediction using these data produced a linear correlation with *r* ^2^ equal to 0.99 (Cozzini et al., 2002), indicating that when pH values of solution and structure data match each other, the HINT prediction is remarkably good.

**FIGURE 3:**
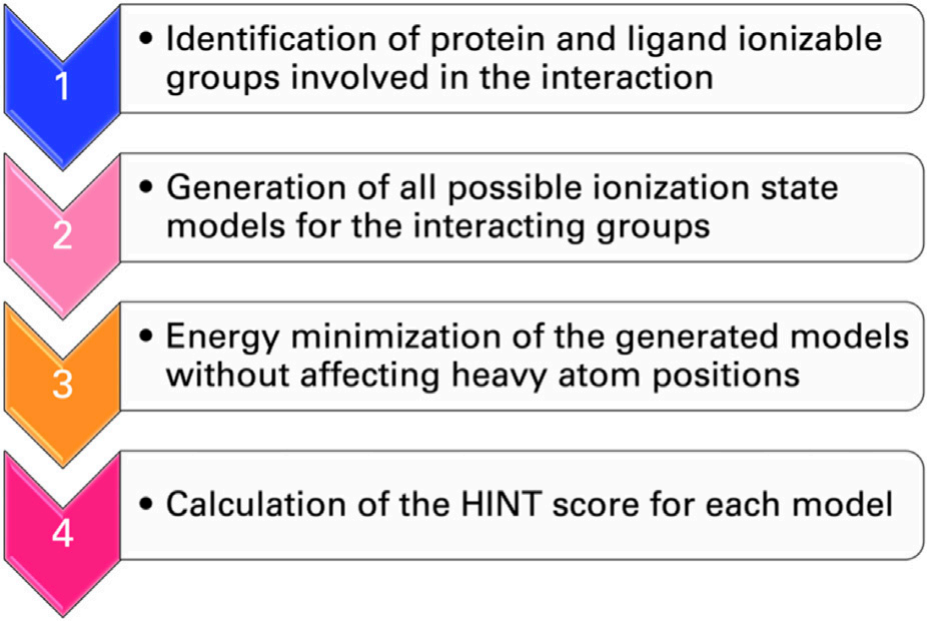
Flowchart of the computational titration.

To address, in a more general way, the dependence of the HINT score on the protonation state of interacting groups of ligand and protein, a protocol called “computational titration” was designed (Fornabaio et al., 2003; Kellogg et al., 2004; 2006; Spyrakis et al., 2004). The steps required by this method (Figure 3) are as follows: i) the identification by a careful inspection of the three-dimensional structures of the ionizable groups of ligand and protein that can contribute to the free energy of binding; ii) the generation of all possible ionization state models for the interacting groups; iii) the energy minimization of the generated models with no movement of the heavy atoms (isocrystallographic models); and iv) the determination of the score for each model with HINT. This is a rather simple problem when only a few ionizable residues or functional groups are involved but increases rapidly in complexity. Each acid has three possible states (unprotonated and protonation at the two oxygens), while each amine or thiol has two, and—if considered—guanidyl could have five. The result is the dependence of the interaction free energy on group ionization states and the identification of the pattern of group ionization states, which leads to the optimal interaction strength. This, in turn, can be correlated with a well-defined pH value (Spyrakis et al., 2004).

We applied the computational titration algorithm to the analysis of the interaction between neuraminidase and nine inhibitors for which three-dimensional structures and inhibition constants were known from the literature (Fornabaio et al., 2003), between HIV protease and its substrate peptide (Spyrakis et al., 2004) and between dihydrofolate reductase and three ligands (Kellogg et al., 2006). In the case of the computational titration procedure for neuraminidase, one proton at a time was introduced into the molecular model. Here, the protein possesses three ionizable amino acid sidechains, namely, Glu 119, Asp 151, and Glu 276, in the active site and one carboxylic moiety on one of the ligands. Thus, a total of four protons were added to the models, plus the starting 0-level model in which all ionizable groups are unprotonated. For each added proton, all possible models were generated, some of them being more chemically probable than others. As shown in Fornabaio et al. (2003), the dependence of HINT score vs. added protons exhibits, not surprisingly, a bell shape, suggesting the optimal protonation state of the ionizable residues, and thus the pH for optimal interaction strength. A further step in the optimization of the computational titration, which requires building and energy minimizing sometimes thousands of models with specific protonation for each protein–ligand complex, was to exclude high-energy models (chemically unplausible) and include a statistical mechanics evaluation of all models. This refined approach was applied to the computational titration of HIV protease and its peptidic substrate (Spyrakis et al., 2004). There are four ionizable residues in the active site, i.e., Asp 25α, 29α, 30α, and 25β, and four potentially interacting ionizable groups on the substrate, i.e., three carboxylates and one amine. In this case, 4,374 unique protonation models were generated, ranging from the most basic with all sites deprotonated (overall charge of −7) to the most acidic with all sites protonated (overall charge of +1). Titration analysis showed: i) a quite large range of HINT scores for models containing the same number of protons indicating favorable and unfavorable proton distributions (Spyrakis et al., 2004), ii) a sharp increase in the HINT score as protons were added that levels off upon five added protons, and iii) a good correlation between experimental data, collected between pH 5 and 3, and the Boltzmann-averaged HINT scores.

### 3.3 Resolution and prediction quality

Another factor that shows a profound impact on the predictions based on HINT, but also to all other codes, is the quality of the three-dimensional structures. The higher the resolution, the lower the standard error in the HINT prediction. We have observed that by selecting within the 53 complexes only structures determined with a resolution better than 2.5 Å, the standard error of the prediction improved to 1.8 kcal/mol compared to 2.6 kcal/mol when the included complexes possess a resolution within 3.2 Å (unpublished data). A better crystallographic resolution leads to a more precise geometry and well-defined distances of interacting groups that impact HINT scores.

Overall, the HINT score for a protein–ligand complex depends, in addition to other contributions, on hydrogen-bonding contribution. A particular property of HINT is that it is very sensitive to the positioning and orientation of hydrogen-bonding protons. It is well known that hydrogen atoms can rarely be detected crystallographically, except for structures with resolutions higher than ∼1 Å. Thus, automated procedures were put in place to insert hydrogen atoms bound to heavy atoms and to correctly orient them to optimize the geometry for hydrogen bonds and, therefore, their strength and scores, without altering the position of the crystallographically determined heavy atoms. The energetic contribution of hydrogen bonds also plays a significant role when water molecules bound to protein active sites and/or ligands are considered (see the following section).

## 4 HINT applied to the evaluation of protein–ligand interactions: the contribution of water molecules

Nothing happens in biological processes without the direct or indirect contribution of water. Protein–ligand complexes, as well as most biological complexes, form in water and often involve the displacement of many water molecules from protein binding sites. However, a few water molecules might be retained that further stabilize the complex association. Water displacement or retention participates in the overall free energy of binding, but the detailed estimation of each water molecule’s contribution is often far from trivial. Therefore, hit identification and, most of all, lead optimization might suffer from the uncertainty of retaining or removing specific water molecules. This has been and continues to be an interesting problem to us, and we have attempted to rationalize water’s role and contribution by means of the HINT code and specifically developed tools (Figure 3), such as rank (Kellogg and Chen, 2004), to address hydrogen-bonding quality, free energy contributions of water–ligand interactions (Fornabaio et al., 2004) and water–protein interactions (Amadasi et al., 2006), the water relevance metric for assessing water conservation with respect to ligand binding (Amadasi et al., 2008), and a tool for similar to GRID (Goodford, 1985) for placing water molecules using relevance (Kellogg et al., 2005).

### 4.1 HINT estimation of the energy contribution provided by bridging and conserved water molecules

**FIGURE 4:**
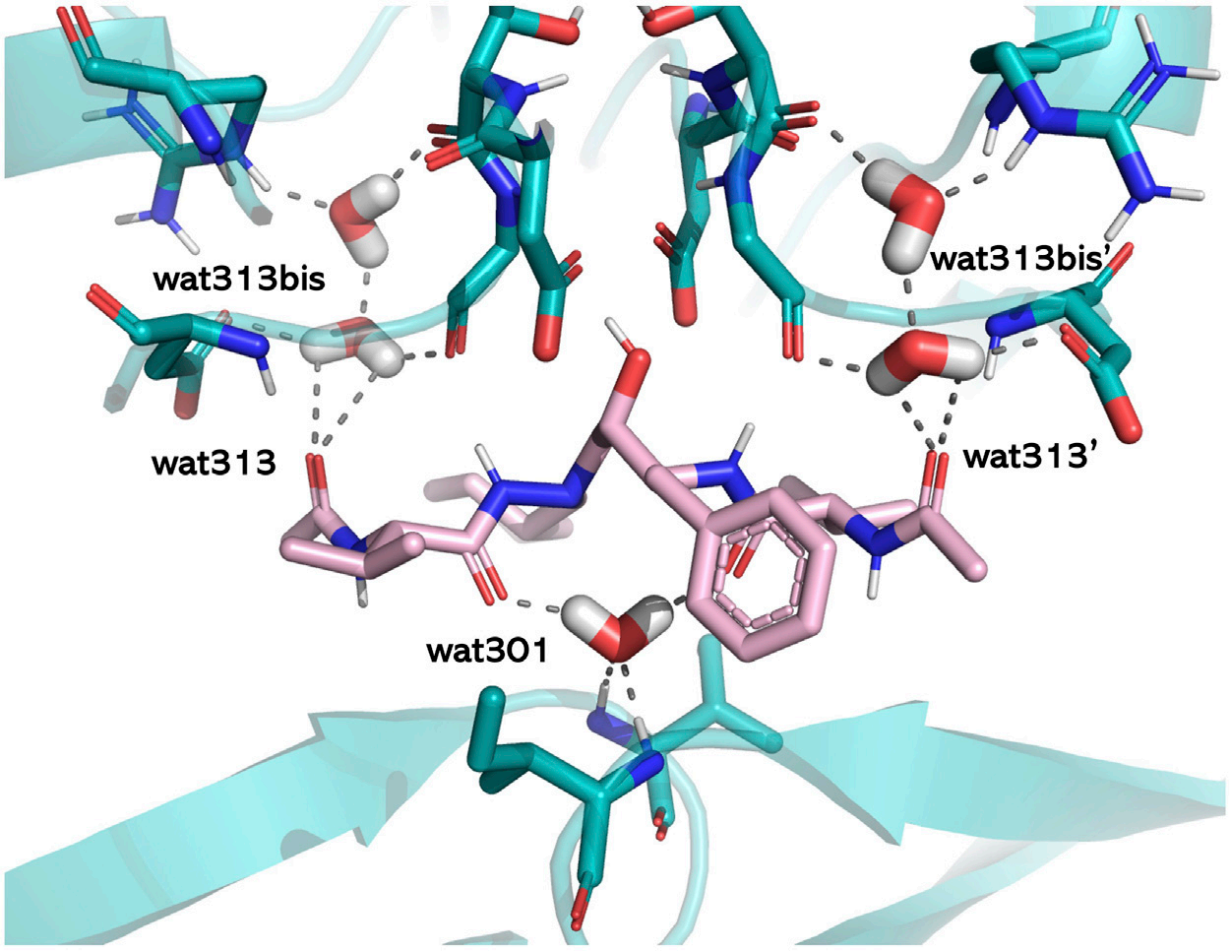
Structural water molecules in the HIV-1 protease binding site. The residues lining the pocket (light blue), the ligand (pink), and the water molecules are shown in capped sticks. The protein is displayed in cartoons, and H-bonds involving water molecules are shown by gray dashed lines (PDB ID: 1HIH). The image has been obtained with PyMol version 2.x.

The literature provides numerous cases in which water plays crucial roles in mediating protein–ligand interactions. Examples are represented by bosutinib binding to Src kinase (Levinson and Boxer, 2014), thermolysin and its water network stabilized by carboxybenzyl-Gly-(PO_2_)-L-Leu-NH_2_−based ligands (Krimmer et al., 2014), the water networks of the adenosine A2A receptor and their perturbations resulting from ligand binding (Congreve et al., 2012), or by the well-known case of HIV-1 protease (Lam et al., 1994). Trying to rationalize the associated water roles, we used HINT to estimate the energetics of binding site waters in 23 HIV-1 protease/inhibitor complexes that belong to the different classes of hydroxyethylenic ligands, peptidomimetic diol derivatives, cyclic ureidic derivatives, and cyclic sulfamide derivatives. The latter pair was specifically designed to displace a conserved water molecule (water 301) in the binding site. A careful analysis of the X-ray structures of the complexes showed the presence of three different structural water categories, that is, water 301, symmetrically located in the binding site with respect to the catalytic one and H-bonding Ile 50 and Ile 150 backbones, waters 313 and 313′, located in a more peripheral region, and waters 313bis and 313bis’, inserted in small clefts in the binding site. The protein–ligand interaction calculated by HINT, with the inclusion of the water contribution, showed that bridging water molecules, such as 301, significantly contributes to the overall energy (about 4–6 kcal/mol) and leads to a better correlation between experimental and predicted free energy. In contrast, the contribution of more peripheral waters is variable or not significant and strongly dependent on the chemical nature and size of the ligand (Fornabaio et al., 2004). However, the binding energy gained by the new ligand-to-protein (Ile 50, Ile 150) interactions in the water 301-displacing cyclic derivatives was very similar to the energy previously associated with the water-to-ligand and water-to-protein interactions (Figure 4). As a conclusion, we recommended including the contribution of water molecules when predicting the free energy of binding but paying specific attention to ensure that this adds information and not just noise.

We implemented the previous work by evaluating not only the energy associated with protein–water interaction but also the geometry quality of the H-bonds formed by each water molecule to obtain a more reliable estimation and prediction of water’s role in mediating protein–ligand association (Amadasi et al., 2006). First, 12 solvated proteins, in both the *apo* and *holo* form complexed with inhibitors, were considered for a total of 2,186 co-crystallized water molecules. Water molecules were classified and separated into the following categories: 1) water molecules in active sites; 2) water molecules deeply inserted into cavities; 3) buried water molecules; 4) first shell external water molecules (within 4 Å of the nearest protein heavy atom); and 5) second shell external water molecules (more than 4 Å from the protein; see the study of Amadasi et al. (2006). Each water molecule was evaluated and classified according to the interactions made with the protein, by means of the HINT score, and to the number of potential H-bonds, by means of the rank algorithm (Kellogg and Chen, 2004).

### 4.2 The Rank algorithm

The latter can evaluate the count, strength, and geometry of potential hydrogen bonds for each water molecule in the cognate structure and is also used to optimize water position and hydrogen placement. Rank can vary from 0, for waters not involved in any hydrogen bonds, to around 6, for waters forming four high-quality hydrogen bonds in terms of bond length and angle geometry, and it is calculated by the following equation:

$$Rank=\underset{n}{\sum }\left\{\left(2.80\;Å/r_{n}\right)+\left[\sum_{m}\cos\left(\Theta_{Td}-\Theta_{nm}\right)\right]/6\right\},$$ (3)

where *r*_*n*_ is the distance between the water oxygen and the target heavy atom; *Θ*_*Td*_ is the ideal angle of 109.5°, and *Θ*_*nm*_ is the angle between targets. Also, any angle less than 60° is rejected, as well as the corresponding bond.

The analysis showed that both HINT and Rank scores increase when evaluating water molecules in the second and first hydration layers, to waters in active sites, up to waters in protein cavities, or completely buried in the protein matrix.

Then, 15 protein–ligand complex binding sites, in which the presence of at least one bridging water molecule was reported in the literature, were analyzed to provide reference HINT scores and Rank values for bridging waters. We observed that the average Rank for protein–water and ligand–water was estimated to be 3.0 and 1.5, respectively, suggesting that proteins are better able to embed bridging waters than ligands, which is quite reasonable considering the residue sidechain flexibility and the presence of clefts and dips in active sites. We have to consider that a Rank equal to 3 might correspond to three H-bonds but also to two very good bonds in terms of distance and geometry. However, no significant difference was provided in terms of the HINT score for protein–water and ligand–water interactions.

Finally, we analyzed a set of nine proteins in both native and complexed state and classified water molecules in active sites in the following categories according to their relevance: 1) conserved water molecules in binding sites bridging protein–ligand interaction; 2) conserved water molecules in binding sites not relevant for protein–ligand interaction; 3) conserved water molecules in binding site cavities; 4) conserved water molecules in peripheral binding site regions; 5) water molecules displaced by ligands replacing their function, i.e., functionally displaced; 6) water molecules displaced by ligands only occupying their room, i.e., sterically displaced; and 7) missing waters. Water molecules with Rank >1.5 and HINT score <150 can be considered as sterically displaceable and, thus, potentially removable by ligands at moderate cost. Water molecules having, instead, Rank values in the 1.5–4.0 range and HINT scores >150 can be more relevant and quite likely retained with respect to drug design, while water molecules with Rank >4 are too buried in the binding site and of less interest (Amadasi et al., 2006).

### 4.3 The Relevance metric

The diagnostic potential of the HINT score and Rank in predicting displaceable, bridging, or buried water molecules was further implemented in the more quantitative Relevance metric (Amadasi et al., 2008). Although water molecules are usually excluded from consideration in structure-based drug design, the rapid identification of Relevant water molecules that might be retained or displaced by *ad hoc* designed ligands (any water can be, in principle, displaced) can significantly affect the energetics of protein–ligand interaction. By using a training set of thirteen proteins, crystallized in the native and complexed state, with a total number of 125 water molecules, we combined information coming from the HINT score and Rank in the following equation, describing the overall probability (*P*_*A*_) of specific water to be Relevant:

$$P_{A}=\frac{{P_{R}\left(\left|W_{R}\right|+1\right)}^{2}+{P_{H}\left(\left|W_{H}\right|+1\right)}^{2}}{{\left(\left|W_{R}\right|+1\right)}^{2}+{\left(\left|W_{H}\right|+1\right)}^{2}},$$ (4)

where *P*_*R*_ and *P*_*H*_ are calculated by nonlinear polynomial regressions and correspond to the percent probability for conservation based on the Rank and HINT score, respectively, while *W*_*R*_ and *W*_*H*_ are the weights of Rank and HINT score probabilities, respectively (Amadasi et al., 2008). This analysis showed Rank and HINT score values associated with conserved waters: *P*_*R*_ > 60%, corresponding to Rank >2.3, and *P*_*H*_ > 80%, corresponding to HINT score >400. Otherwise, *P*_*H*_ > 40%, although corresponding to HINT score >100, was considered indicative of non-conservation. When applied on a test set of nine native and complexed proteins, for which specific bridging water molecules were previously identified, 59 of 68 water molecules were correctly predicted, corresponding to a success rate of 87%, which increased to 92% when only water molecules from X-ray structures having a resolution <2.0 Å were considered. Importantly, the crystallographic B factors, often used in such analyses did not improve this model.

### 4.4 Hot and cold water

In a later contribution, we designated water molecules in protein matrices as “cold” and “hot” according to their internal energy and possible displacement (Spyrakis et al., 2017). Hot water can also be considered “unhappy” water, that is, not stable in binding sites or at protein surfaces, because of the presence of extensive hydrophobic regions. Being unhappy, they would benefit from being displaced toward a more polar environment as the bulk, where they could maximize the number of H-bonds and provide a favorable variation of the binding free energy. Cold water, being stably bound in polar environments can be, instead, considered as “happy” molecules able to extend protein/ligand functions and to participate in the protein structure, dynamics, and function. Their displacement might not be at a trivial expense and may be associated with meaningless or even unfavorable variation of the binding free energy.

Behind the hot/cold classification is, in fact, their enthalpic and entropic contributions. Several considerations can be drawn that might be useful with respect to a drug design perspective (Ahmed et al., 2013; Spyrakis et al., 2017): i) hot water molecules in binding sites can be easily displaced with a manifest gain in entropy and a negligible loss in enthalpy; ii) attention must be paid when displacing cold water molecules since the entropic gain might not balance the enthalpic loss; iii) the enthalpy/entropy compensation associated with cold water displacement can lead to quite small changes in the binding free energy, thus often making drug design efforts ineffectual; iv) hot water networks around protein–ligand complexes hide hydrophobic moieties stabilizing the association; and v) both hot and cold water molecules at the protein–protein interface can provide precious indications when designing protein–protein inhibitors.

### 4.5 Virtual screening with the HINT scoring function

Clearly, the emphasis on modern drug discovery has shifted over the past decade or so toward higher-throughput modeling approaches, such as virtual screening. Before using HINT for extensive docking experiments, we were curious whether docking scores from various scoring functions correlated better with RMSD (root-mean-squared distance) or free energy of binding. In other words, does reproducing a crystal structure by docking, which is how most scoring functions are optimized, necessarily produce accurate predictions of binding energy? We examined 19 protein–ligand complexes for which X-ray crystallographic structures and binding energy data were available, and we calculated experimental vs. computationally-derived free energy correlation by means of the HINT free energy scoring tool and other scoring functions (Spyrakis et al., 2007a). Correlations drawn after re-docking the cognate ligands in their corresponding protein structures were generally better with the HINT scoring function (Spyrakis et al., 2007a). Also, it was obvious from this study that scoring functions uniquely calibrated for the dataset or sets under study should nearly always be preferable to universal scoring functions.

The HINT scoring function described earlier has indeed been a powerful tool for virtual screening (Salsi et al., 2010; Farzan et al., 2011; Spyrakis et al., 2014; Obaidullah et al., 2018; Kayastha et al., 2022) with a few caveats, including: 1) the scoring function is extremely sensitive to structure, especially properly optimized hydrogen-bonding interactions that include the donor hydrogen’s position, and 2) while it is more than fast enough for scoring docking models, it is not, at present, in the “giga” docking timescale range. Thus, HINT, in our hands, has been used as a second scoring function after docking with programs like GOLD or AutoDock and their native scoring functions have been applied as primary filters. In this use case, our in-house studies generally have been relatively small-scale and speculative, i.e., purchasing 10–100 compounds for actual assay with a variety of low-to-medium-throughput methods. Nevertheless, about one-in-five of the compounds identified by virtual screening with HINT scoring were active.

Three examples of this research are as follows: 1) 14 pentapeptides, binding to *Haemophilus influenzae* O-acetylserine sulfhydrylase (OASS) with affinities ranging from μM to mM, were identified with good correlation between computational and experimental data, excluding peptides bearing a positive charge, which are likely overestimated by HINT. These results, combined with X-ray structures of the three best complexes, defined a pharmacophoric scaffold for the design of peptidomimetic inhibitors for this enzyme (Salsi et al., 2010). 2) To identify new antimicrobials toward *Treponema denticola* cystalysin, we performed virtual screening on 9,357 compounds with the FLAPsite algorithm (Baroni et al., 2007), docked those most promising, and rescored with HINT in a consensus approach. Among the 17 compounds selected for testing, two showed IC_50_s in the low μM range, identifying interesting hits for the development of novel antimicrobials (Spyrakis et al., 2014). 3) The most recent publication using HINT in this way (Kayastha et al., 2022) details the discovery of three new potent eIF4A inhibitors that diminished the viability of diffuse large B-cell lymphoma (DLBCL) cells. The actives were discovered via target-based virtual screening in the MolPort database, followed by pharmacophore-based and analog screening. Modeling suggests that these compounds clamp eIF4A into mRNA in an ATP-independent manner and depress eIF4A-dependent oncogene expression. Eukaryotic translation initiation factor 4A is essential in translation initiation as it unwinds the secondary structure of messenger RNA upstream of the start codon and enables active downstream ribosomal recruitment.

## 5 HINT applied to the evaluation of protein–protein interactions

Protein–protein interactions are emerging as one of the most important features of biological structures. Building an understanding of their contributions is challenging because of many of the aforementioned issues. However, an important advantage of the HINT model in evaluating protein–protein interactions is that the key hydrophobic–hydrophobic interactions are handled in a robust atom-to-atom manner (Kellogg and Abraham, 2000; Sarkar and Kellogg, 2010), not lumped together as lipophilic surface contact areas or other aggregate metrics. There are two types of protein–protein interactions: the first between distinct separate proteins, which have many implications for biological function, metabolism, and diseases, and the second within a protein—as in protein multimers, protein folding, and intramolecular associations. Although these definitions are broad and probably inclusive of most biology, we have applied HINT to several specific problems as we looked for insight into the link between structure and function.

### 5.1 Dissecting the energetics of protein–protein associations

While protein–ligand interactions are complex on their own, the addition of more degrees of freedom when two proteins interact is another level of complexity. For example, in a typical protein–ligand interaction, there will likely be only a few relevant water molecules, but at a protein–protein interaction surface, there can be one or two dozen. Using the aforementioned computational titration algorithm (Fornabaio et al., 2003; Spyrakis et al., 2004; Kellogg et al., 2006) would typically involve 2–3 ionizable residues or functional groups in a protein–ligand complex, but again, at a protein–protein surface, this could be a much larger number.

**FIGURE 5:**
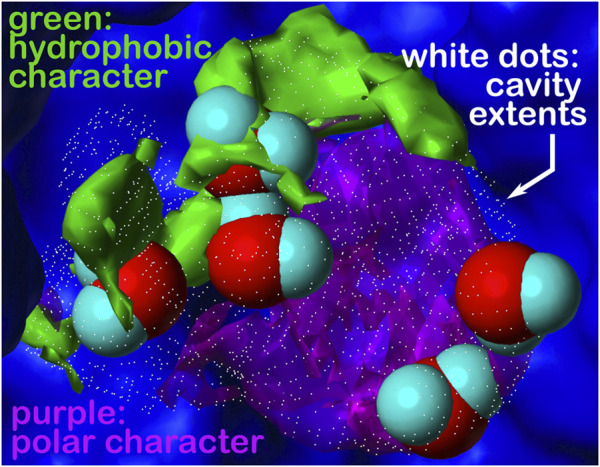
Cavity is formed during the association of the human placental RNase inhibitor (hRI) and human angiogenin (hAng) proteins to form the complex (pdbid: 1a4y). A closeup view of a portion of this inter-protein interface is shown. The cavity’s extents are depicted by rendering in white dots; the green and purple contours represent the character of the proteins surrounding that cavity, hydrophobic and polar, respectively. What we are terming a “hydrophobic bubble” is found in the upper left region of the cavity as it encloses or traps three non-relevant waters in a largely hydrophobic (green) environment, where their strongest interactions may be amongst themselves. The other two waters in the cavity are in a polar (purple) region and are more relevant. See the study of Ahmed et al. (2011) for a further discussion on water molecules at protein–protein interfaces. Note that displacement of the three non-relevant “hot” waters, likely as a cluster, may reveal a pocket large enough for targeting as a site for protein–protein inhibition.

To explore the water issue, we adapted the rank and relevance algorithms to protein–protein interactions in order to probe the roles of water molecules found specifically at protein–protein interfaces (Ahmed et al., 2011; 2013). The result was the surprising conclusion that less than one-quarter of waters found at such interfaces were engaged in truly structure-supporting bridging roles, and just over one-quarter of them had unfavorable interactions with respect to both proteins. The remainder, over one-half, had favorable interactions with one protein and unfavorable interactions with the other (Ahmed et al., 2011). We proposed a new structural motif—the hydrophobic bubble—to describe the phenomenon of one or a collection of water molecules “trapped” in a hydrophobic cavity formed by the protein–protein association (Figure 5). Moreover, we hypothesized that these bubbles are critical to protein function as points/regions of instability to assist in protein–protein dissociation when needed. Two implications of these observations are worth mentioning: 1) it will be very difficult to predict the presence of water in such loci based only on energetics as they are by definition unstable, although water clusters do gain enthalpic advantage through their interactions with each other, and 2) these phenomena may present a unique opportunity for drug discovery at protein–protein interfaces as they are the true “hotspots” (Spyrakis et al., 2017).

**FIGURE 6:**
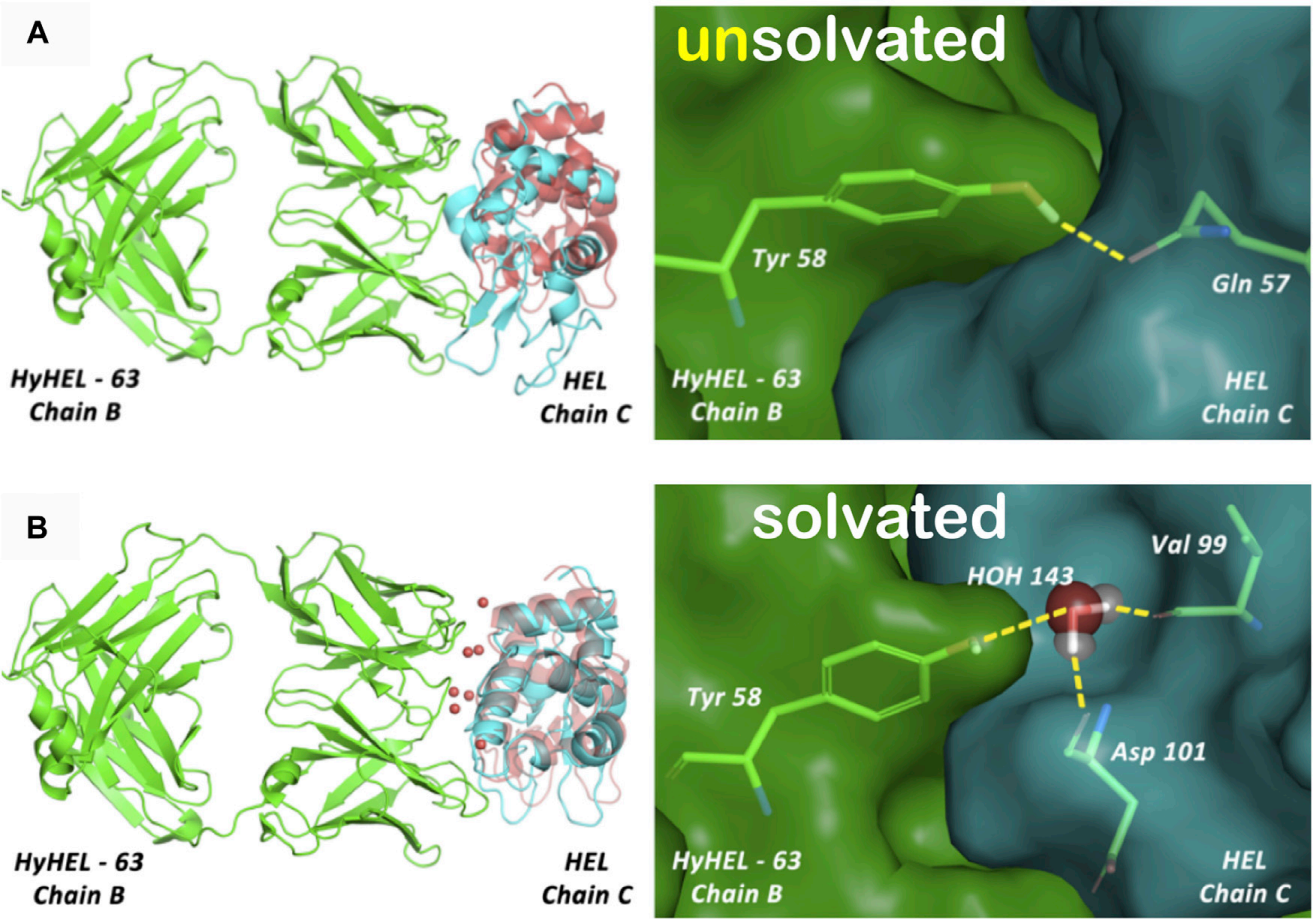
**(A)** Unsolvated docking results for the HyHEL-63/HEL complex. The left panels overlay the predicted ligand poses (cyan) with the crystal structure (red), and the right panels illustrate the interactions of B/Tyr58 with ligand residues. This model is representative of 40% of generated poses found after clustering of the complete set of docking solutions but does not show native residue–residue contacts. **(B)** Solvated docking results for the HyHEL-63/HEL complex. This model shows native water-mediated residue-residue contacts, with B/Tyr58 showing a water-mediated hydrogen-bonding network with C/Val99 and C/Asp101 (see the study of Parikh and Kellogg, 2014).

To further examine our insistence on the importance of water at protein–protein interfaces, we reported a docking study on a small number of protein–protein complexes both “dry”, as allowed by the native ZDOCK (Pierce and Weng, 2008; Pierce et al., 2011) program, and “wet”, where we tricked ZDOCK into recognizing interfacial water molecules (Parikh and Kellogg, 2014) (Figure 6). The latter models were far superior by HINT score and numerous metrics developed by the Critical Assessment of PRedicted Interactions (CAPRI) communitywide experiment (Janin et al., 2003; Mendez et al., 2005), but creating such models was very tedious at that time due to the limitations inherent in the ZDOCK code, e.g., its automatic deletion of anything that was not in its library of 20 amino acids. This, coupled with the inherent problems in predicting the presence of water molecules that are “not” energetically viable, although obviously present, set us in a different direction with respect to HINT-based structure prediction (section 5.3), including protein–protein docking.

### 5.2 The intramolecular HINT score

As we were developing HINT, implementing an intramolecular score was a very simple addition. However, it was not immediately obvious how to apply it to interesting and important problems until we started thinking about the difficulties of dealing with low-resolution crystal structures and how their reflection data are collected and refined. When the resolution is high, the reflection data are more than adequate to model the structure within the electron density envelopes and features, such as hydrophobic interactions, are seen. However, at low resolution, somewhat crude molecular mechanics forcefield algorithms are applied, and hydrophobic interactions—that are not explicit in these force fields—are often lost. We used the intramolecular HINT score as an adjuvant to contemporary structure refinement protocols (Koparde et al., 2011). We showed that low-resolution structures refined by including our HINT intramolecular score-based protocol were significantly more native-like based on structure quality metrics.

More recently, Agosta et al. (2022) used the intramolecular HINT score to evaluate the stability of proteins in response to single (and multiple) site mutations. In particular, the SARS CoV-2 spike glycoprotein that exhibits interaction between its receptor-binding domain and the human angiotensin-converting enzyme 2, and has been seen to mutate rapidly with devastating consequences, was modeled and mutated *in silico*. The intramolecular HINT scores for the alpha, beta, gamma, delta, and omicron variants confirm that all mutated trimeric spike protein structures are similarly or more stable than the wild-type. In addition, these scores show that the receptor-binding domains of these mutants are more stable than the wild-type. The HINT intramolecular scoring is very rapid, and presuming the structures are well-modeled is suggestive that it may be useful as a pre-screening tool for as-yet undiscovered mutants for any protein.

### 5.3 Protein structure predictions and 3D hydropathic networks

**FIGURE 7:**
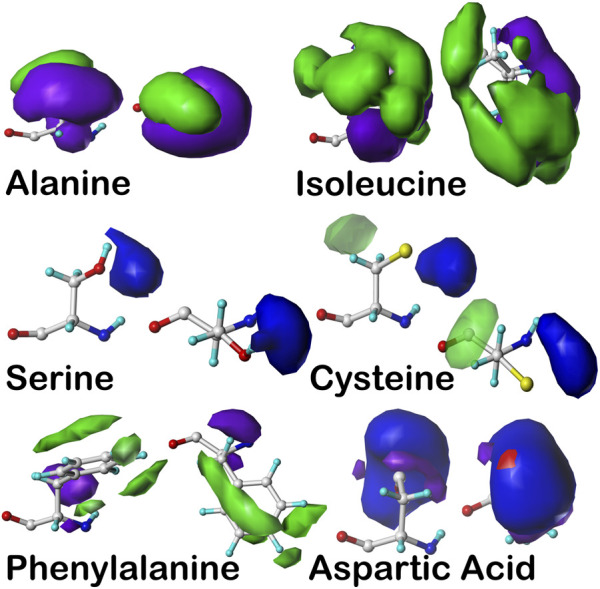
Example contoured hydropathic interaction basis maps for six residue sidechain types. The full set for all residue types includes about 18,000 such maps. Each of these maps illustrate one observed collection of interactions—discovered by 3D map clustering—between the named residue and its environment, including all other residues and water. Each map is taken from the set calculated in the same alpha helix region of the Ramachandran plot, and all are contoured at largely similar iso-density levels. Two views are plotted for each case: left- the CA–CB (z) axis is pointed up, and right- the CA–CB axis is pointed out of the page. The interaction types are color-coded by type: green- favorable hydrophobic interactions, i.e., depicting hydrophobic interactions between the residue depicted and other atoms in its environment; purple- unfavorable hydrophobic (i.e., hydrophobic-polar) interactions; blue- favorable polar (e.g., hydrogen bonding) interactions; and red- unfavorable polar interactions. For more explanation, see the following: alanine- Ahmed et al. (2019), isoleucine- AL Mughram et al. (2023), serine and cysteine- Catalano et al. (2021), phenylalanine- AL Mughram et al. (2023), aspartic acid- Herrington and Kellogg (2021).

The concept of interaction networks has been recognized for decades as it is a way to systematize protein structure, especially regarding hydrogen bonding. Our work with HINT has continually highlighted the parallel and often more important hydrophobic interactions. For example, it is not a coincidence that the α1β1 (and α2β2) interface of hemoglobin are largely unaffected by the deoxy to oxy hemoglobin transitions (and dominated by hydrophobic contacts between the two subunits), while the α1β2 interface, with just as many hydrogen bonds, has few hydrophobic interactions (Abraham et al., 1997) and shifts significantly in the transition. Also relevant are the “unfavorable” hydrophobic interactions (including those involving water), which are similarly key elements of protein structure. To systematize all of these observations and to create a platform for structure modeling, we developed a 3D interaction mapping paradigm wherein all interactions (polar and hydrophobic, favorable, and unfavorable) between a residue and its structural environment are visualized as contours with specific colors, strengths, and positions for each residue in a protein (Ahmed et al., 2015; Ahmed et al., 2019). To control backbone conformation, we adopted a chess square schema in which the Ramachandran plot is binned into 8 × 8, 45° × 45° chess squares (Ahmed et al., 2015). Further binning or parsing by sidechain conformations were applied as follows: *χ*_1_ for asparagine, aspartic acid, cysteine, histidine, isoleucine, leucine, methionine, phenylalanine, proline, serine, threonine, tryptophan, and tyrosine, and *χ*_1_ and *χ*_2_ for arginine, glutamine, glutamic acid, and lysine. The former, thus, have three parses per chess square and the latter nine (AL Mughram et al., 2021). These maps can, interestingly, after binning, be clustered into limited sets of interaction motifs characteristic of residue type, solvent accessibility, and structural role. The number of clusters obtained is dependent on the population of the associated bin and the complexity of observed and anticipated interactions. For example, alanine sidechains show about four unique maps per chess square, while arginine can show as many as 18 maps in some of the nine *χ*_1_/*χ*_2_ parses. Altogether, there are about 18,000 maps for our soluble proteins data set—each represents a unique constellation of interacting atoms for that residue type, backbone conformation, and sidechain parse. For illustration, Figure 7 sets out example contoured maps for six diverse residue types, all in the α-helix region of the Ramachandran plot. Importantly, it is not the identity of those interacting atoms but their specific properties, e.g., hydrophobic, polar, hydrogen bond donor or acceptor, *etc.*, and water molecules can fill these roles just as other amino acid residues, cofactors or small molecules can. Because these roles are agnostic in terms of sequence, we call this paradigm “3D interaction homology” to emphasize that it is not the residue identity but its interactions that drive the structure.

**FIGURE 8:**
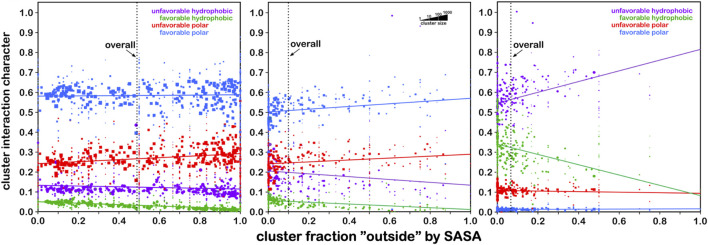
Residue interaction character as a function of solvent-accessible surface area. Each data point represents a cluster of interaction maps. The size of each marker is representative of the number of residues within that cluster (see legend). Left: the character of interactions made by serine residues are dominated (∼60%) by favorable polar (blue) with very minor contributions from hydrophobic interactions (green) that decrease from ∼5% at low solvent accessibility to near zero at fully exposed. On average, serine’s solvent exposure is around 50% (vertical line). Center: same graph for cysteine. The overall trends are quite similar, except that, on average, cysteine’s solvent exposure is only ∼10%, indicating that cysteine is far more likely to be found buried in a protein than on its surface. Right: same graph for S–S bridged cysteine (cystine), where the average solvent exposure is now only about 7%. Also, the character of interactions made by cystine is dominated by unfavorable hydrophobic interactions (purple), followed by favorable hydrophobic. Thus, -S–S- bridged cysteines are found most frequently in strongly hydrophobic environments that are buried. These data may provide insight into predictions of cysteine -S–S- bridge formation in protein structures (see the study of Catalano et al., 2021).

With this extensive set of in-hand data, ∼18,000 maps abstracted from ∼750,000 residues in our dataset, we have a complete, quantifiable picture of the hydropathic valence of each residue type and its potential roles in a protein’s hydropathic interaction network. Exploiting this set of maps involves “matching” each backbone-aligned map’s encoded interactions between residues. Because there are limited sets of such maps for residue type and backbone conformation, this is a comparatively simpler problem than *de novo* atomistic minimization. More importantly, however, using HINT scoring and its core interaction model has enabled our view of the structure to be built upon a free energy framework and to account for some more subtle features of interactions that are not necessarily detected by molecular mechanics-based approaches. For example, sidechain maps of aromatic residues, phenylalanine, tyrosine, and tryptophan, show evidence of pi–pi stacking and pi–cation interactions (AL Mughram et al., 2021). Also, the computational titration algorithm was applied to aspartic acid, glutamic acid, and histidine map generation and allowed pH tuning of those residues’ interaction profiles (Herrington and Kellogg, 2021). Likewise, an understanding of the large differences in structural role between the serine and cysteine was explored with our HINT/interaction maps approach (Catalano et al., 2021). While our first goal was to document the interactions in which these residues engage, we were also able to develop an improved understanding of their roles in protein structure: 1) serine is considered to be somewhat more polar than cysteine but is *significantly* more solvent exposed; 2) while both have similarly consistent interaction roles (∼50–60% favorable polar, Figure 8) regardless of their accessibility, clearly very few cysteines are found on the outside of proteins; 3) it is also interesting (Figure 8) that bridging (-S–S- bonded) cysteines exist in much more hydrophobic environments than their unbridged analogs, which may be mechanistically suggestive. In another more recent study, the aliphatic hydrophobic residues were examined in both soluble and membrane proteins (AL Mughram et al., 2023), which surprisingly revealed somewhat modest differences in interaction profiles for these residues in soluble proteins, where they are often buried, and membrane proteins, where they are exposed to lipids. The latter is particularly important because very few X-ray crystal or even cryo-EM structures of membrane proteins retain their native lipids (Guo, 2020).

In the last few years, AlphaFold2 and RoseTTAFold (Baek et al., 2021; Jumper et al., 2021) achieved an astonishing power of prediction of protein three-dimensional structures thanks to the application of deep learning methods that revolutionized the approach applied until then (Goulet and Cambillau, 2022) This new capacity has changed the landscape of structural biology and drug discovery in surprisingly fundamental ways. There remain many proteins poorly predicted by these tools, and the emerging information concerning membrane proteins has likely not been effectively incorporated in these algorithms, as those structures themselves are not well-understood, especially with respect to the role(s) of their native lipids. In some cases, the value of experimental structural characterization has even been questioned! It should be noted, however, that even if computational predictions generate accurately-folded protein models, they are not likely to produce models that have sidechain conformations accurate enough for structure-based drug discovery and design (Tong et al., 2021). Indeed, it is the subtle features of the structure that drive toward success in these endeavors. HINT and other modeling tools that focus on the subtle interactions beyond gross folding and salt bridges will continue to be relevant and useful.

## 6 HINT applied to the evaluation of DNA–ligand and DNA–protein interactions

The inclusion in HINT of both hydrophilic and hydrophobic terms makes it able to predict energy contributions in DNA–ligand and DNA–protein complexes very well. Indeed, it is well known that the structure of DNA is stabilized by the stacking interactions between the planes of the nucleobases along the helix axis, which are the main factor in stabilizing the double helix (Yakovchuk et al., 2006), and by the hydrogen bonds between complementary base pairs. Stacking interactions are hydrophobic in nature, whereas hydrogen bonds are essentially hydrophilic (Feng et al., 2019); therefore, when other (macro)molecules bind to DNA, their interactions are conditioned by both contributions assessed by the HINT.

### 6.1 Intercalation agents

The analysis of energetic interactions involving DNA by using HINT was exploited for the first time in the study of the effects of antineoplastic drugs, such as chlorambucil (an alkylating agent), and anthracycline antibiotics, such as doxorubicin, daunorubicin, and others (which act as intercalating agents). In the first example, HINT analysis of a computational model of the adducts derived by administration of chlorambucil to the shuttle vector plasmid pZ189 demonstrated that the methylenes, as well as the phenyl ring of this drug, can form favorable hydrophobic interactions with nucleotides near the adduct site in the minor groove of DNA, promoting alkylation at the N-3 position of adenine (Wang et al., 1994).

Next, HINT was used to study the selectivity of doxorubicin intercalation and binding in all 64 unique base pair quartet combinations. The results showed that the interactions between doxorubicin and the base pairs above and below the intercalation site are mainly polar and favorable, resulting from acid–base interactions between the heteroatoms of the antineoplastic drug and the nucleotide bases. In addition, favorable hydrophobic contributions are also present. Specificity was mainly associated with hydrogen bonds, in particular with a base pair three positions away from the intercalating site (Kellogg et al., 1998). These analyses were subsequently extended to explore the binding of six different anthracycline antibiotics (doxorubicin, daunorubicin, hydroxydoxorubicin, 9-dehydroxydoxorubicin, adriamycinone, and daunomycinone) to 32 different DNA octamer sequences. The analysis showed that the differences in free energy among the various compounds were in line with that experimentally observed, indicating that HINT was able to pinpoint the energetic contributions of ligand functional groups most relevant to sequence specificity (Cashman et al., 2003). A further investigation of 65 doxorubicin analogs and their complexes with eight octamer DNA sequences allowed prediction of the net energetic contribution of several functional groups in the tested ligands and detection of selective ligands for the different combinations of DNA sequences simulated in this study (Cashman and Kellogg, 2004).

Analysis of the interactions of the two antibiotics gentamicin and paromomycin with 12 designed analogs with ribosomal RNA also showed that rings III and IV of these compounds are involved in important polar interactions with rRNA (Cashman et al., 2001). Therefore, HINT has proven to be a sensitive tool for dissecting interactions between nucleic acids and their ligands and for assessing the strength of interactions with existing drugs, as well as for predicting possible modifications of these molecules to improve their affinity and/or selectivity.

### 6.2 Interactions between proteins and DNA

HINT was tested for its ability to evaluate interactions of much more complex systems, such as protein–DNA complexes. An initial study was conducted on the interactions between estrogen receptors (ER) alpha and beta and the corresponding estrogen-responsive elements (EREs) near estrogen-regulated genes (Marabotti et al., 2007). By analyzing the structure of the DNA-binding domain (DBD) of ERα and the homology-based model of ERβ bound to the ERE sequence, it was possible to identify the residues that contributed most to the binding affinity. Furthermore, by mutating each nucleotide pair in the two halves of the ERE binding site with all other possible pairs, it was possible to understand how mutations in the different positions of ERE could affect the binding affinity in both complexes. The results showed that, consistent with the experimental results, ERα binds the consensus ERE sequence with higher affinity than ERβ and that few amino acids and bases of the consensus sequences are involved in specific interactions. Specifically, HINT was able to discriminate with high sensitivity the affinity of ERα/ERβ DBDs for ERE sequences, as well as for non-ERE sequences used as negative controls (glucocorticoid- and progestinic responsive elements), whereas DDNA (Zhou et al., 2005), another predictor of protein–DNA interaction energies available at that date, did not. We hypothesized that the reasons for this failure was that the DDNA predictor was based on a knowledge-based statistical potential trained on a reference database not including protein–DNA complexes. However, it is significant that the HINT code was not derived specifically from the analysis of DNA structures; therefore, this fact confirmed us the general validity of the hydropathic approach (Marabotti et al., 2007).

For this particular set of complexes, the specificity of protein–DNA sequence binding did not appear to be much affected by water molecules. However, given the importance of the contribution of water in the thermodynamics of DNA–protein recognition, this investigation was extended in parallel to 39 additional DNA–protein complexes for which a three-dimensional structure was available (Spyrakis et al., 2007b). Thus, it could be shown that the inclusion of water molecules at the interface between protein and DNA (the so-called “bridging waters”) in the energetic contribution calculated by HINT improved the correlation between its score and the experimental free energy of association, with a lower standard error. The fraction of bridging waters in this set of experiments was only 3.5% of the water molecules detected in the 39 crystallographic complexes, consistent with the percentage of water mediating recognition between proteins and DNA identified previously (Reddy et al., 2001). It was also possible to observe that the orientation and binding strength of these water molecules depended more on the nature of the amino acid sidechain than on the type of DNA bases.

### 6.3 Protein–DNA recognition

**FIGURE 9:**
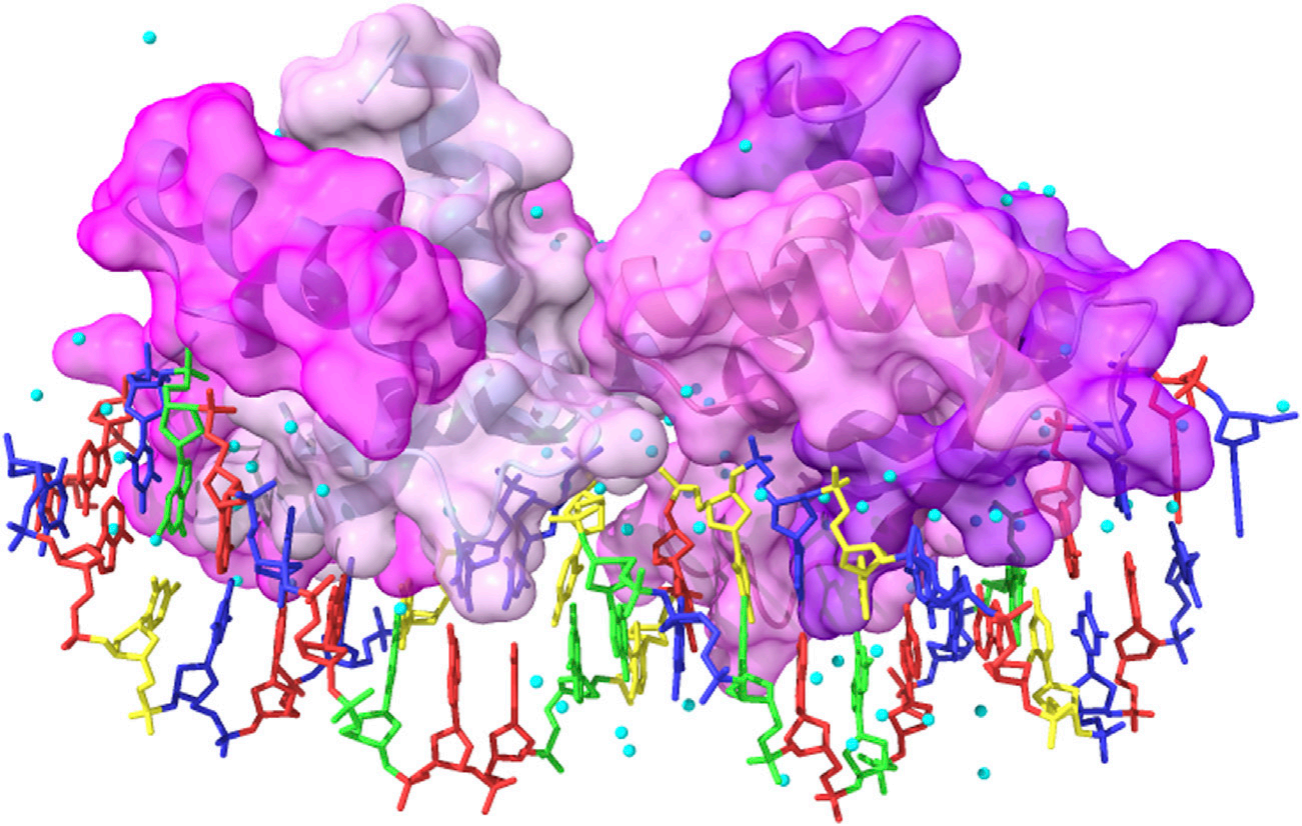
Complex between the wild-type gene-regulating protein ARC and the DNA (PDB ID: 1BDN). The four chains of the protein are represented with different shades of pink and with highlighted solvent-accessible surface area. In transparency, it is possible to see the secondary structure elements composing the protein. The color code for the nucleotides is as follows: A: red, T: blue, G: green, and C: yellow. Cyan balls represent water molecules. The image has been obtained with UCSF ChimeraX (version 1.5).

A more comprehensive study on energy-based prediction of the specific recognition between amino acid residues and nucleotide bases was subsequently performed on a dataset of 100 high-resolution protein–DNA complexes (Marabotti et al., 2008) (Figure 9) and used to predict specific contacts between amino acids and nucleotide bases in a set of 45 zinc finger–DNA complexes identified by phage display selection (Ghosh et al., 2006). By applying the HINT code, three main questions could be answered: i) Which amino acid–base pairs are energetically most relevant to achieve a specific interaction? ii) Are there energetic propensities that justify specific recognition of a nucleotide base by an amino acid? iii) Are bridging waters able to influence the specificity of amino acid–base recognition? The results showed that the amino acids that interact most frequently with nucleotide bases are Arg, Asn, Lys, Gln, Thr, Ser, Asp, and Gly; in fact, HINT calculated that the sum of their contacts accounts for more than 70% of the total number of contacts. Arg-G, Asn-A, Asp-C, Gln-A, Glu-C, and Lys-G appear to be the most energetically favorable contacts (Arg-G being the interaction that accounts for about 2/5 of the total HINT score for the complexes), while Asn-T, Asp-G, Gln-T, Glu-G, Ile-T, Leu-T, Met-T, and Val-T are unfavorable interactions. The analyses also showed that the same amino acid–nucleotide base pairs relevant to protein–DNA interactions are also particularly involved in water-mediated interactions.

**FIGURE 10:**
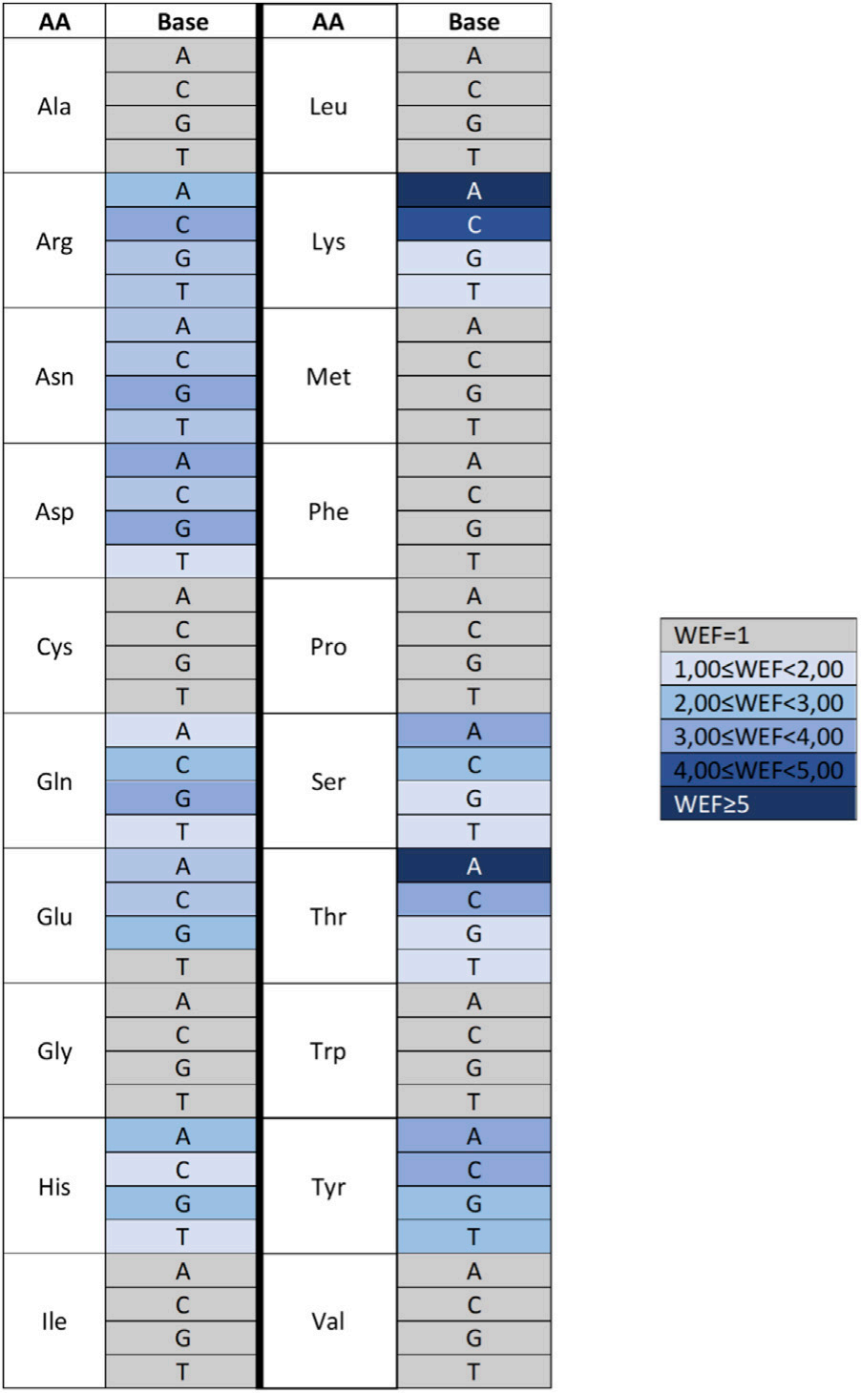
Heat map describing the water enhancement factor (WEF), i.e., the HINT score enhancement due to water contribution, calculated for each amino acid–base pair. A water enhancement factor of 1 indicates an amino acid (AA)–base (B) interaction with no significant bridging water molecules. Data are extracted from Marabotti et al. (2008).

For some amino acid–base pairs, it was also possible to calculate a “water enhancement factor,” that is, the ability of bridging waters to enhance the energetics of the amino acid–base interaction (Figure 10). Finally, based on the HINT score extracted from this analysis, it was possible to correctly predict more than 70% of the experimentally observed amino acid–base pairs in the zinc finger–DNA used as a test set. This percentage increased to nearly 90% when a relevance-weighted success descriptor, considering the relative energy relevance of each amino acid–base pair to the total protein–DNA recognition energy, was included. In this way, it was possible to show that amino acid–nucleotide base preferences could be explained by the energy-based analysis performed by HINT better than through qualitative approaches based on purely geometric considerations. Moreover, HINT also made it possible to predict unfavorable interactions, some of which are surprisingly well-conserved and usually involve the methyl group of thymine. This finding is interesting in that one might speculate that the amino acid–base interaction evolved before DNA development and was later adapted to DNA, but the presence of the thymine methyl group still continues to be a disruptive element in protein–DNA interaction (Marabotti et al., 2008).

## 7 Conclusion

The prediction of events in antiquity was a very profitable but risky job, from the prophet Cassandra to those relying on Sibilla oracle cards. In addition to gambling, which, by definition, remains unpredictable, the forecast of weather is perhaps the most common modern-day testing ground for prediction. In use are algorithms that take into account the many variables that dictate sunny or rainy days, windy or calm weather conditions, and temperature. In this case, the robustness of the prediction can be and is verified every day, and the applied algorithms are constantly adjusted with incremental but beneficial improvements.

Prediction of the binding affinity between a protein and a ligand, or for any two biological molecules, independently of their molecular weights, is as challenging to predict as a protein structure. Multiplicity and diversity are the rules as many small energetic contributions lead to either loose, medium, or tight complexes. Some of these contributions are difficult to pinpoint as they deal with entropy or other emergent phenomena. HINT is one of the few codes that have attempted energetic evaluations of the molecular events associated with the formation of a protein–ligand complex—considering both enthalpic and entropic contributions—in a very simple and “natural” way (Kellogg and Abraham, 2000). This idea was one of many innovative and transformative concepts that can be credited to Donald J. Abraham. He was an outstanding medicinal chemist who, by having visited Max Perutz’s laboratory at the Cambridge MRC (Medical Research Council), had a clear appreciation of the value of protein structural information for the discovery, design, and development of novel drugs.

The results of applications of HINT to many diverse protein–ligand and protein–nucleotide complexes with and without water contributions, reported herein, demonstrate that it is possible to obtain very usable, if not accurate, predictions of protein–ligand strength in short times and even with very low computational power. This paves the way for the design of chemical entities that correctly fit within protein active sites, enabling either inhibition or enhancement of their function, and potentially act as drugs to treat diseases. This was the dream of Abraham, and we are still pursuing it. We might be a bit closer!
